# How do classroom environment and grit influence foreign language academic achievement: the mediating roles of foreign language anxiety and boredom

**DOI:** 10.3389/fpsyg.2026.1887486

**Published:** 2026-09-04

**Authors:** Ruishu Wang, Shiyong Jiang, Yuqian Liu, Rui Wang

**Affiliations:** 1 Foreign Studies College, Northeastern University, Shenyang, China; 2 College of Arts, Northeastern University, Shenyang, China; 3 School of Humanities and Law, Northeastern University, Shenyang, China; 4 College of Information Science and Engineering, Northeastern University, Shenyang, China

**Keywords:** classroom environment, foreign language academic achievement, foreign language anxiety, foreign language boredom, grit

## Abstract

**Introduction:**

The study aims to explore the influence mechanism of classroom environment and grit on foreign language academic achievement of college students, and focuses on the mediating roles of foreign language anxiety and foreign language boredom.

**Methods:**

Through 409 valid questionnaires, a mediation model was constructed. The influence pathways of variables and the mediating effects of foreign language anxiety and foreign language boredom were examined and analyzed by SPSS, AMOS and 5000 bootstrap samples.

**Results:**

The findings reveal that: (1) both the classroom environment and grit can directly predict learners’ academic emotions and foreign language academic achievement; (2) foreign language anxiety and foreign language boredom can significantly and directly predict foreign language academic achievement; (3) academic emotions mediate the relationship between classroom environment, grit, and foreign language academic achievement, with the mediating effect of foreign language boredom being stronger.

**Conclusion:**

The results of the study show that classroom environment and grit not only directly promote foreign language academic achievement, but also indirectly affect foreign language academic achievement through the mediating effect of foreign language anxiety and foreign language boredom. These conclusions provide theoretical support and practical insights for optimizing foreign language classroom instruction and enhancing learners’ academic performance.

## 1 Introduction

Foreign language learning is not merely a process of knowledge acquisition. It is a comprehensive activity in which learners engage in cognitive construction, emotional experiences, and behavioral participation within specific instructional contexts. Currently, there are several issues in foreign language classroom teaching practices. For instance, some learners’ motivation for foreign language learning is primarily driven by external factors (e.g., test-taking), while the deep and enduring intrinsic motivation of them is lacking (Liu et al., 2024). Additionally, challenges such as teacher-centered instruction, passive student engagement, and inadequate classroom environment have triggered negative emotions like anxiety that subsequently impact learners’ academic performance (Cheng and Ping, 2025).

Previous research has predominantly focused on academic achievement, language proficiency, and teaching strategies. However, it insufficiently addressed learners’ perceptions of their classroom environments, personal characteristics such as grit, and academic emotions. According to Luan and Quan (2025), positive personality traits, emotions, and classroom environment could affect second language acquisition. As the primary setting for language learning, classroom environment characterized by teacher support, peer interaction, and task design directly influence students’ learning experiences (Ma et al., 2024). Grit, as a positive personal trait, aids learners in maintaining motivation when confronted with challenges (Teimouri et al., 2022; Mikami, 2024). Academic emotions, including anxiety and boredom, act as mediators that connect external environments with personal characteristics, exerting a substantial influence on learners’ academic performance, achievement levels, and competency development (Xiao and Zhang, 2024). Furthermore, according to the control-value theory, grit and classroom environment can affect learners’ control appraisal and value appraisal from internal and external dimensions, triggering various academic emotional responses that ultimately impact foreign language achievement. As foreign language teaching research shifts its focus from outcomes to processes, the significance of classroom environment, grit, and academic emotions in foreign language education has become increasingly prominent. However, the relationships among these factors and their specific pathways influencing foreign language academic achievement have yet to be fully elucidated.

To address the research gap, our study integrates classroom environment (external context), grit (internal personal trait), academic emotions, and foreign language academic achievement into a unified analytical framework based on control-value theory, aiming to achieve two research objectives. The first research aim is to examine the pathways through which classroom environment, grit, foreign language anxiety, and foreign language boredom influence foreign language academic achievement. The second objective is to test the potential mediating roles of foreign language anxiety and foreign language boredom in these relationships. The findings not only enhance the research perspectives of the control-value theory in the context of foreign language education, but also offer practical implications for foreign language instruction and learning support.

Our study demonstrates innovative value in two aspects. First, by integrating classroom environment, grit, academic emotions, and foreign language academic achievement into a unified analytical model, it compares the differential impacts of external environmental factors and internal personal characteristics on learners’ foreign language academic achievement. The finding not only provides empirical evidence for optimizing teaching environment and adjusting instructional strategies, but also expands the explanatory dimensions of the control-value theory within the context of foreign language learning. Second, by elucidating the internal mechanisms of negative academic emotions, it compares the strengths of the mediating effects of foreign language anxiety and foreign language boredom on academic achievement. It not only enriches the observational perspective of research on foreign language learning emotions, but also lays a theoretical foundation for further exploration of the impact mechanisms of high arousal emotion (anxiety) and low arousal emotion (boredom) on foreign language learning in future studies.

## 2 Theoretical framework and literature review

### 2.1 Control-value theory

The control-value theory plays a significant role in the field of second language acquisition and emotion research (Wang and Hui, 2024; Pekrun, 2006). According to the core tenets of the theory, academic emotions encompass a variety of emotional experiences that are directly linked to academic activities or outcomes. Pekrun et al. (2002) demonstrated that learners can experience a range of emotions while engaging in academic activities, anticipating academic outcomes, or reflecting on past academic results, including enjoyment, hope, pride, anger, anxiety, shame, hopelessness, and boredom. The theory effectively elucidates the reasons behind the diverse emotional experiences of second language learners throughout the learning process by examining the interaction between perceived control and value. Furthermore, it clarifies the specific impacts of these emotional experiences on learning outcomes (Pekrun et al., 2006).

The control-value theory posits that academic emotions arise from individuals’ appraisals of their perceived control and value related to academic achievement. The theory emphasizes that learners’ appraisals of control and value regarding specific learning tasks, along with their intrinsic factors (e.g., grit) and external environmental factors (e.g., classroom environment) can significantly influence learners’ emotions. Furthermore, academic emotions can directly or indirectly affect the execution of academic activities and the attainment of academic success (Pekrun, 2006; Pekrun et al., 2023). For instance, learners with grit are capable of investing persistent effort in academic endeavors and maintaining consistent interest. Typically, learners with higher levels of grit have greater control over their academic activities and possess a more pronounced perception of their value. The state fosters positive emotional experiences and enhances learners’ academic performance. Conversely, learners with lower levels of grit often lack the motivation to sustain prolonged effort and exhibit relatively weaker interest in academic activities. As a result, their appraisals of control and value of academic activities remain at lower levels, leading to negative emotions and the decline in academic achievement. Another example is that positive classroom environment can enhance learners’ control and value appraisals of academic activities, foster positive emotions, effectively stimulate their learning motivation, and ultimately improve academic performance. Conversely, if the perceived classroom environment of learners’ is unfavorable, their control and value appraisals of academic activities will decrease correspondingly, leading to negative emotions and a decline in academic achievement. Therefore, it can be asserted that learners’ individual characteristics and environmental factors influence the generation and variation of academic emotions by affecting learners’ control and value appraisals. Furthermore, academic emotions can impact the formation of academic outcomes by influencing various psychological processes, such as cognition and motivation (Pekrun, 2006). Consequently, it can be concluded that emotions serve as mediating roles between internal factors, external factors, and learning outcomes (Pekrun, 2006).

### 2.2 Factors affecting academic achievement in foreign language learning

#### 2.2.1 Classroom environment

Classroom environment refers to the comprehensive state formed in the classroom setting, encompassing the overall atmosphere, situational tone, and emotional environment (Dorman et al., 2006). The research dimensions include classroom belongingness, class cohesion, fairness, and mutual support between teachers and students (Fraser, 2002). Researchers have identified several typical characteristics of positive classroom environment, which are specifically manifested as: outstanding cohesion among students, ample support provided by teachers, deep engagement of learners in classroom activities, emphasis on inquiry-based learning, clear task orientation in classroom activities, collaborative and mutually supportive peer relationships, and the maintenance of fairness in classroom evaluation and treatment (Fraser et al., 1986; Li et al., 2021).

Our study adopts the framework proposed by Peng and Woodrow (2010), focusing on three dimensions of classroom environment: teacher support, student cohesiveness, and task orientation. Specifically, teacher support refers to the assistance provided by teachers, the friendly attitudes, and the trust they place in students. Student cohesiveness denotes the extent to which students understand each other and offer mutual help and support. Task orientation pertains to students’ perceptions of the importance of completing learning tasks, maintaining focus on task topics, and recognizing the practical utility of these tasks (Peng and Woodrow, 2010). The classroom environment is a crucial factor influencing the learning process and outcomes, encompassing various elements such as teacher-student interactions, instructional methods, and supportive classroom settings. It plays an indispensable role in shaping students’ learning experiences and facilitating their academic achievements (Li and Dewaele, 2021). Numerous studies have been conducted to verify the phenomenon. Majumder and Beri (2025) performed a systematic analysis of 243 empirical research papers regarding the impact of classroom climate on English academic achievement. The results indicate that the positive classroom environment fosters emotional security, promotes effective teacher-student interactions, establishes clear classroom rules, and maintains well-organized instructional arrangements. A high quality classroom climate can significantly enhance students’ English proficiency and positively influence their overall academic performance. Furthermore, Majumder et al. (2025) found that classroom climate had a significantly and directly effect on English achievement. In contrast, Zheng and Feng (2023) found that the none of the components of perceived classroom environment directly influenced English achievement. But two dimensions of perceived classroom environment indirectly influence the English achievement by foreign language boredom. In summary, it can be concluded that the classroom environment significantly affects students’ foreign language achievement, directly or indirectly. The study will specifically verify and explore the mechanism of influence.

#### 2.2.2 Grit

The concept of grit, proposed by Duckworth et al. (2007), was defined as an individual’s persistent attitude and passionate state toward long term goals. The construct comprises two sub-dimensions: perseverance of effort and consistency of interest. Perseverance of effort refers to an individual’s inherent tendency to sustain effort toward goals over long term development, while consistency of interest denotes the ability to maintain a stable and enduring passion for higher-order goals when the learners face various challenges, obstacles, or setbacks (Duckworth et al., 2007; Lee, 2022). Learners’ higher levels of grit are more likely to develop stronger learning motivation, demonstrate greater resilience, and achieve greater academic success in their learning activities (Duckworth et al., 2007; Fathi et al., 2026). Numerous empirical studies have validated the correlation between grit and academic achievement. For instance, Harpaz et al. (2024) investigated the relationship between grit and academic achievement with a sample of 351 college students. The results indicate that grit serves as both a predictor and a mediating factor for academic achievement. Given the significant impact of grit on academic achievement, many researchers have incorporated the related concepts of grit into the field of second language acquisition research. Teimouri et al. (2022) defined grit in the context of second language learning as L2 grit, characterizing it as the perseverance of effort and consistency of interest demonstrated by learners throughout the language learning process. The study also demonstrated the significant impact of L2 grit on second language academic achievement. Relevant research has confirmed that L2 grit plays a crucial role in learners’ attainment of language learning accomplishments. For instance, Li and Yang (2024) found a positive correlation between L2 grit and learners’ second language learning achievement. Additionally, Zhao and Wang (2023) conducted an empirical study with a sample of 504 Chinese secondary school students aged 12–16 and revealed that grit served as a significant predictor of both emotions and learning achievement in language learning. Furthermore, Zhao et al. (2024) verified that grit could effectively predict language achievement through foreign language enjoyment. Meanwhile, Botes et al. (2025) conducted a study with 182 English summer course participants from three private language institutes in Iran with an average age of 19.89 years and identified L2 grit as the dominant predictive factor influencing language learning achievement.

However, there are some different research findings in academia. For instance, Khajavy et al. (2021) conducted a study with 1,178 students enrolled in general English courses at two universities in Iran and found no significant correlation between various dimensions of learners’ grit and foreign language achievement. And these dimensions could not predict foreign language performance as well. Additionally, Yamashita (2018) conducted an empirical study with a sample of 78 students, which revealed no significant relationship between the dimensions of grit and academic language achievement. Based on the previous research findings, we assert that the importance of grit in language learning has gained widespread recognition. However, there is currently no unified consensus of the functional relationship between grit and foreign language academic achievement. Furthermore, whether the relationship is moderated or influenced by other factors remains to be thoroughly explored and validated through subsequent research.

#### 2.2.3 Foreign language anxiety

Foreign language anxiety is a kind of situational anxiety that encompasses a unique set of self-perceptions, beliefs, emotions, and behavioral responses associated with the specific learning difficulties encountered in classroom language learning (Horwitz et al., 1986). It is characterized as a negative emotional experience marked by worry and tension that arises in the process of foreign language learning and its practical application (MacIntyre, 1999). Based on the control-value theory, Li and Li (2023) further conceptualized foreign language anxiety as an academic emotion within foreign language learning contexts that was negative, highly activated, and outcome-related. Thus, foreign language anxiety, as a distinct type of situational anxiety, refers to the negative emotional state experienced by learners when they are placed in a specific foreign language learning environment and are required to use the foreign language to complete various learning tasks.

As a key factor influencing learning outcomes (Dewaele et al., 2023a), the role and impact of foreign language anxiety on academic achievement have garnered significant attention from researchers. Some scholars have revealed a substantial negative correlation between foreign language anxiety and foreign language academic achievement based on the meta-analytic methods (Teimouri et al., 2019; Dikmen, 2021; Xiao and Zhang, 2024). Concurrently, multiple empirical studies have further validated the adverse effects of foreign language anxiety on academic performance in foreign language contexts. For instance, Jin et al. (2024) conducted an empirical study involving 203 Chinese college students, revealing a moderate negative correlation between the levels of foreign language learning anxiety and learners’ self-perceived foreign language performance. Meanwhile, Hu et al. (2024) surveyed 631 students and found significant negative correlations between language achievement and various dimensions of foreign language anxiety. Additionally, other research indicates that foreign language anxiety can serve as a predictor of learners’ foreign language achievement (Liu, 2023; Dewaele et al., 2023b; Li and Xing, 2024).

However, previous research shows divergent findings regarding the impact of foreign language anxiety on academic achievement in foreign language learning. For instance, Nasution et al. (2022) surveyed 41 students of grade 11 and found a significant positive correlation between anxiety and learners’ English achievement (*p* < 0.05, *r* = 0.98). Additionally, Luo and Xiong (2025) conducted an empirical analysis with a sample of 476 learners, revealing that neither facilitative anxiety nor debilitative anxiety could significantly predict learners’ foreign language academic achievement. Overall, current academic research on the impact of foreign language anxiety on foreign language academic achievement has yet to reach a consistent conclusion. The study aims to conduct specific empirical research on the unsolved issue.

#### 2.2.4 Foreign language boredom

In the process of foreign language learning, boredom is a prevalent subjective psychological experience among learners. It is characterized by a lack of interest in language learning tasks and related activities, insufficient willingness to actively participate, and an inability to derive adequate stimulation from these tasks (Pawlak et al., 2020). The phenomenon is specifically defined as a low arousal, disengaged psychological state experienced by learners in ongoing language learning activities. The state results from a lack of interest in learning, insufficient classroom engagement, or interference from external negative situational factors (Li et al., 2023). Furthermore, it represents a negative emotional experience encountered by learners in the process of foreign language acquisition (Pawlak et al., 2022).

In recent years, the research topic of foreign language boredom has gradually gained prominence within the academic landscape (Pawlak et al., 2025; Shimray and Wangdi, 2025). Researchers have undertaken empirical investigations to explore the relationship between foreign language boredom and foreign language learning. Relevant findings indicate that boredom negatively affects learners’ engagement in foreign language learning (Dai and Wang, 2024) and detracts from learning outcomes (Ma et al., 2025; Li et al., 2025), ultimately leading learners to disengage from the language learning process and adversely impacting their language performance and overall learning (Dewaele and Botes, 2025). Research on the influence of foreign language boredom on academic achievement in language learning has yielded relatively consistent conclusions within the academic community. For instance, Li et al. (2025) conducted a meta-analysis examining the overall correlation between boredom and second language learning performance, confirming a negative association between the two variables. Similar conclusions were reached in the study by Huang and Zhang (2025). Furthermore, previous research has demonstrated that foreign language boredom significantly predicts learners’ academic achievement in foreign language learning. For instance, Wu and Kang (2023) conducted an empirical analysis involving a sample of 235 English learners, revealing a significant negative correlation between boredom and English academic achievement. Their findings indicate that the emotional state can directly predict English academic performance. Similarly, Dewaele et al. (2023a) surveyed 502 Moroccan EFL learners with an average age of 22.37 years and confirmed that foreign language boredom served as a predictor of foreign language academic achievement.

However, some studies have reached divergent conclusions. For instance, in a study involving 159 students conducted by Dewaele et al. (2025), correlation analysis revealed a negative relationship between academic performance improvement and foreign language boredom; however, multiple regression analysis indicated that foreign language boredom was not a significant predictor of learners’ oral proficiency improvement. Overall, the research on the impact of foreign language boredom on foreign language achievement has yet to reach a consistent conclusion. And our study aims to empirically explore the issue.

### 2.3 The roles of foreign language anxiety and foreign language boredom between classroom environment/grit and foreign language academic achievement

The control-value theory (Pekrun, 2006) provides a theoretical framework for analyzing the formation mechanisms and influence pathways of emotions in foreign language learning, specifically foreign language anxiety and boredom. The theory systematically elucidates the antecedents and consequences of emotions, categorizing emotional antecedents into distal and proximal levels. Distal antecedents include individuals’ internal and external factors, such as personality traits and classroom environment, whereas proximal antecedents pertain to individuals’ judgments of controllability, perceptions of intrinsic value, and evaluations of extrinsic value appraisal. Furthermore, the theory delineates the subsequent effects of emotions, highlighting the indirect impact on academic performance through their influence on psychological and behavioral processes, including cognitive activities, learning motivation, learning engagement, and strategy selection (Pekrun, 2006). It underscores the mediating role of emotions between the antecedent conditions and subsequent outcomes. Relevant research by Li et al. (2023) indicated that foreign language anxiety and boredom were prevalent academic emotions in foreign language learning, and the core principles of the control-value theory can be effectively utilized to interpret and analyze these emotions.

Previous research has validated significant associations between antecedent variables (classroom environment and grit) within the control-value theory framework and their impact on foreign language academic emotions, as well as the subsequent relationship between these emotions and academic performance. Specifically, Li and Dewaele (2021) found that learners with higher levels of grit and more positive classroom environment reported lower levels of foreign language classroom anxiety. It means that increased grit and supportive classroom atmosphere effectively alleviate anxiety in English learning. Furthermore, research by Li and Xing (2024) demonstrated a negative correlation between foreign language anxiety and academic achievement. They found that foreign language anxiety was a significant predictor of academic performance. Additionally, a study conducted by Lyu (2025) involving 40 students revealed a significant negative correlation between grit and boredom in second language learning contexts. The finding suggests that learners with higher levels of grit are more adept at employing strategies such as goal-setting and self-monitoring to mitigate boredom in the learning process. Moreover, boredom has been shown to negatively correlate with second language academic achievement (Li et al., 2025), indicating that boredom can adversely affect learning outcomes. Meanwhile, Wu and Kang (2023) demonstrated that boredom served as a predictor of foreign language academic achievement.

The aforementioned studies have confirmed correlational patterns between the antecedent variables (classroom environment and grit) involved in the control-value theory and foreign language academic emotions (foreign language anxiety and foreign language boredom), as well as the correlational patterns between these academic emotions and foreign language academic achievement. Specifically, as upstream influencing factors, the antecedent variables can trigger the emergence of foreign language anxiety and boredom, which in turn exert corresponding effects on learners’ foreign language academic performance. However, the specific mediating pathways through foreign language anxiety and boredom have not been sufficiently examined.

### 2.4 Hypothesis and hypothesized model

Current academic research has explored the relationships among classroom environment, grit, foreign language academic emotions (foreign language anxiety and boredom), and foreign language academic achievement, respectively, which lays the foundation for further in-depth studies. Firstly, the classroom environment, as a significant external influencing factor, can directly or indirectly predict learners’ foreign language academic achievement by shaping teachers’ characteristics and classroom task design. Secondly, grit, regarded as an individual trait of learners, effectively predicts learners’ foreign language academic achievement through sustained effort and stable learning interest. Thirdly, according to the control-value theory, both foreign language anxiety and foreign language boredom can negatively impact and serve as predictors of foreign language academic achievement. Additionally, the two emotions mediate the relationship between the antecedent variables (classroom environment and grit) outlined by the control-value theory and foreign language academic achievement.

**Figure 1:**
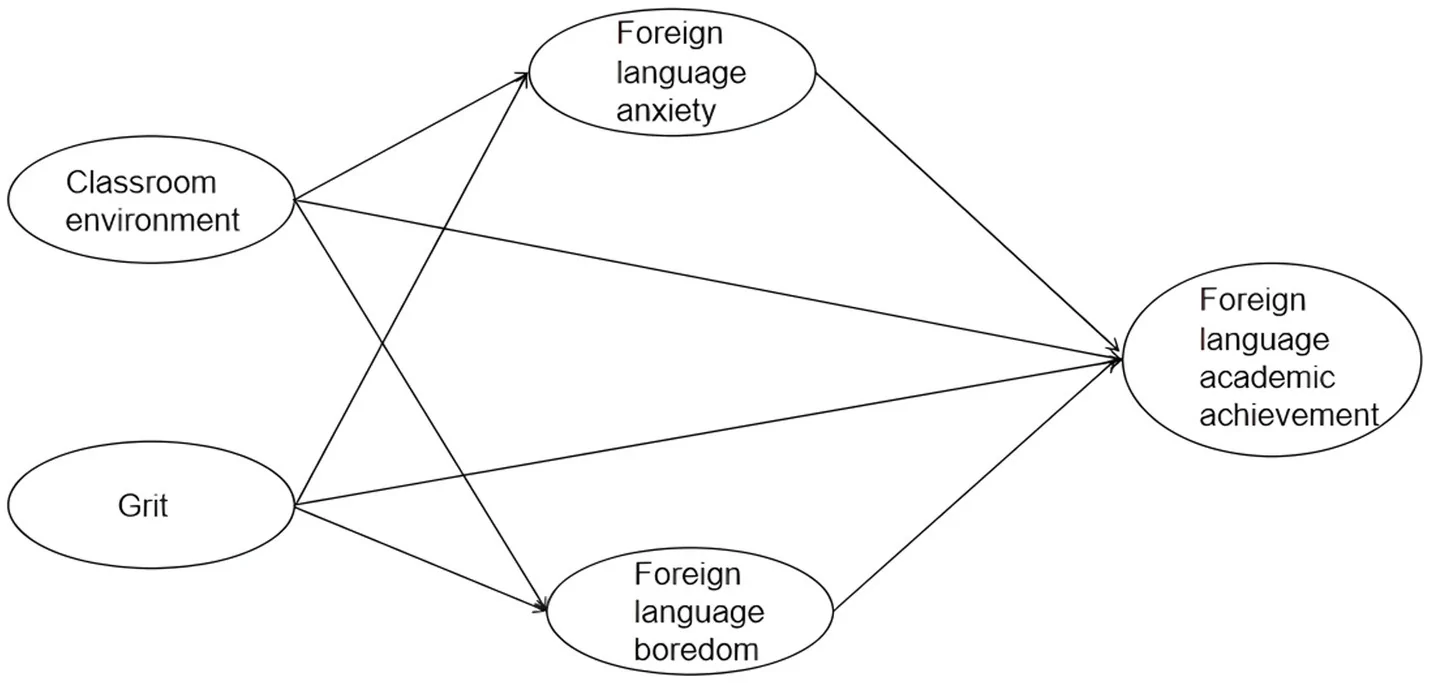
The hypothesized model.

Based on the systematic review of the relevant literature, it can be concluded that current empirical explorations in academia regarding the aforementioned research content have not yet reached a unified consensus. Thus, the study aims to address the following two research questions. RQ1: Can classroom environment, grit, foreign language anxiety, and foreign language boredom significantly predict learners’ academic achievement in foreign language learning? RQ2: Do foreign language anxiety and foreign language boredom serve as significant mediators between classroom environment/grit and foreign language academic achievement, respectively? To address these research questions, the study constructs a hypothesized relational model (as shown in Figure 1) that illustrates the relationships among classroom environment, grit, foreign language anxiety, foreign language boredom, and foreign language academic achievement. The following research hypotheses are proposed.

Hypothesis 1 states that both classroom environment and grit can directly predict foreign language academic achievement. Hypothesis 2 posits that foreign language anxiety and foreign language boredom can directly predict learners’ foreign language academic achievement. Hypothesis 3 suggests that foreign language anxiety and foreign language boredom serve as mediators between classroom environment/grit and foreign language academic achievement.

## 3 Research methodology

### 3.1 Research ethics

The study was conducted in accordance with the Declaration of Helsinki. The research team strictly adhered to the established ethical review procedures, ensuring that informed consent forms were personally signed by all study participants. Prior to conducting the research, all participants were clearly informed about data confidentiality protocols and explicitly acknowledged their right to voluntarily participate in the study and withdraw at any time during the research process.

### 3.2 Participants

The study recruited 503 full-time undergraduate students from three universities in China. To effectively control for key confounding variables, the study ensured homogeneity in participants’ linguistic backgrounds and learning trajectories. All participants were native Mandarin speakers. All subjects began formal English language course in elementary school and have accumulated at least 10 years of English learning experience. In addition, the sampling scope of this study is limited to non-English majors in college public English courses, covering eight disciplinary categories: engineering, science, economics, law, management, literature, education, and arts.

In the classroom acquisition model for Chinese university students learning English as a foreign language (EFL), learners lack natural extracurricular language input and authentic communicative contexts, making their foreign language learning dependent on classroom resources and in-class task experiences. Therefore, the classroom environment becomes a significant variable influencing learners’ academic emotions and achievement in foreign language learning. Within the learning context, grit plays a crucial role in helping students sustainably cope with long-term language learning pressures, overcome negative emotions, and maintain consistent engagement. Furthermore, previous research has shown that foreign language anxiety and boredom among Chinese university students significantly hinder English knowledge acquisition and language proficiency development. These characteristics of Chinese EFL learners provide contextual plausibility and support for the directional relationships between the variables presupposed in this study. Therefore, the subjects are suitable for investigating the mechanisms linking classroom environment, grit, academic emotions, and academic achievement.

Twenty participants withdrew from the study or failed to complete the questionnaire. A total of 483 questionnaires were collected for the study. After data purification, 74 invalid questionnaires were eliminated, resulting in 409 valid responses with an effective recovery rate of 84.68%. Among the valid samples, there were 290 male participants, accounting for 70.90%, and 119 female participants, representing 29.10%. All research variables were measured by 5-point Likert scales. Participants’ foreign language academic achievement was assessed based on their latest final examination scores in English courses. The scores were standardized and converted into a quantitative indicator of subjects’ academic achievement.

### 3.3 Research instruments

The study utilized questionnaires as the primary research tool for collecting quantitative data. The questionnaire comprises two sections. The first section examines participants’ demographic information, including gender, grade, academic discipline, and their final examination scores of English course. The second section focuses on assessing participants’ classroom environment, grit, and foreign language academic emotions (foreign language anxiety and foreign language boredom). All items were rated from 1 (“strongly disagree”) to 5 (“strongly agree”). To ensure participants could accurately understand all items in the questionnaire, the research team conducted a localized translation of the original English scale. The first author initially translated the English scale into Chinese, then submitted the draft for review by the other authors. Each author provided specific suggestions for revisions regarding wording, reading fluency, and semantic accuracy. Through collective discussions, the entire research team systematically refined minor expression deviations in the translated draft, ultimately finalizing the Chinese version of the questionnaire.

#### 3.3.1 Classroom Environment Scale

The study utilized an adapted version of the Classroom Environment Scale developed by Peng and Woodrow (2010). The scale consists of three sub-dimensions: teacher support, student cohesiveness, and task orientation. Twelve items were selected to create a measurement scale appropriate for the study, aimed at evaluating participants’ classroom environment. It encompassed teacher support (4 items), student cohesiveness (4 items), and task orientation (4 items). Representative items include: “The teacher is patient in teaching” “I help other class members who are having trouble with their work” and “Activities in this class are clearly and carefully planned”.

#### 3.3.2 Grit Scale

The measurement of grit was conducted by the L2-Grit Scale which was developed by Teimouri et al. (2022). It was designed for language learning contexts. The scale primarily assesses learners’ sustained commitment and behavioral tendencies in pursuing long term language learning goals, and demonstrates distinct domain specificity. Comprising nine items, the scale includes two sub-dimensions: perseverance of effort (five items) and consistency of interest (four items). Representative items include: “Now that I have decided to learn English, nothing can prevent me from reaching this goal” and “I have been obsessed with learning English in the past but later lost interest”.

#### 3.3.3 Foreign Language Classroom Anxiety Scale

The study employed an adapted version of the Foreign Language Classroom Anxiety Scale developed by Jiang and Dewaele (2019) for measurement purposes. The selected scale comprises eight items from the original scale, which was initially developed by Horwitz et al. (1986), resulting in a more concise version aimed at measuring learners’ physiological manifestations of anxiety, psychological states of nervousness, and lack of confidence in foreign language classrooms. For the current research, six items from the Foreign Language Classroom Anxiety Scale (Jiang and Dewaele, 2019) were selected to construct a tailored measurement scale, which was subsequently utilized to assess participants’ levels of foreign language anxiety. Representative items include: “I always think that the other students speak English better than I do” and “I start to panic when I have to speak without preparation in English Listening and Speaking class”.

#### 3.3.4 Foreign Language Boredom Scale

Learners’ foreign language boredom was assessed by the subscale from the Foreign Language Learning Boredom Scale developed by Li et al. (2023). The subscale comprises eight items. Representative items include: “I start yawning in English class because I’m so bored” and “I am only physically in the classroom, while my mind is wandering outside the English class”.

#### 3.3.5 Foreign language academic achievement

The study utilized standardized scores from participants’ latest final English course examinations as the primary metric for assessing their foreign language academic achievement. The grade data were collected via online questionnaires, with participants self-reporting their scores. The raw examination scores (on a 100-point scale) were converted into standardized Z-scores by SPSS 26.0, referred to “foreign language academic achievement.” The standardized values were subsequently employed in further analyses to represent the foreign language academic performance within the overall research sample.

### 3.4 Data collection and analysis

The researchers distributed online questionnaires to participants via the Wenjuanxing online platform. Prior to participating in the survey, all participants signed informed consent forms. A brief overview of the study was provided in the first section of the questionnaire including two commitments. The first one is that the entire response process will adhere to the principle of anonymity. The second one is that all response data will remain strictly confidential.

Data statistical analysis was conducted by SPSS 26.0 and AMOS 24.0. Firstly, the reliability and validity of the measurement scales were assessed. Subsequently, descriptive statistics and correlation analyses were performed to examine the relationships between the research variables. Then, the study adopts a two-step analytical strategy of “holistic modeling followed by focused testing.” First, structural equation modeling (SEM) is utilized to conduct global estimation of the full structural model and verify the overall fit of the theoretical framework as well as the path relationships between latent variables. The model fit indices were evaluated according to the criteria proposed by previous research: *χ*^2^/*df* ≤ 2 or 3 (Schreiber et al., 2006), CFI > 0.95 (Schermelleh-Engel et al., 2003), GFI > 0.90 (Schumacker and Lomax, 1996), AGFI ≥ 0.90 (Schermelleh-Engel et al., 2003), SRMR ≤ 0.05 (Hu and Bentler, 1995), and RMSEA ≤ 0.06 (Hu and Bentler, 1999). Afterwards, SPSS PROCESS macro (Model 4) is run separately for classroom environment and grit. With 5,000 bootstrap samples, we precisely quantify the indirect effect of each mediating path along with its 95% confidence interval. The built-in pairwise comparison function of PROCESS is further adopted to directly test the effect difference between the two parallel mediating paths of anxiety and boredom. SEM ensures the theoretical coherence of the model, while PROCESS delivers supplementary information unavailable from SEM outputs, namely precise inference and effect comparison of specific mediating paths. The two analytical methods complement each other, rendering the test of mediating mechanisms more transparent and robust.

In addition, prior to the estimation of the main model, the study conducted independent sample *t*-tests and one-way ANOVA to preliminary examine group differences across three types of background variables. The results revealed that these background variables exerted no significant effects on classroom environment, grit, foreign language anxiety, foreign language boredom, and foreign language academic achievement (*p* > 0.05). Accordingly, they were not included as covariates in the main SEM and PROCESS models.

## 4 Results

### 4.1 The reliability and validity test

The reliability test indicated strong reliability levels for the Classroom Environment Scale. The Cronbach’s alpha coefficients for three subscales and total scale were 0.845 (teacher support), 0.851 (student cohesiveness), 0.806 (task orientation), and 0.921, respectively. Additionally, the Grit Scale exhibited strong reliability. The Cronbach’s alpha coefficients for two subscales and total scale were 0.883 (perseverance of effort), 0.834 (consistency of interest), and 0.915, respectively. Furthermore, the Foreign Language Anxiety Scale demonstrated a commendable level of reliability, with the Cronbach’s alpha coefficient of 0.851. The Foreign Language Boredom Scale also exhibited strong reliability, with the Cronbach’s alpha coefficient of 0.890.

The structural equation modeling test revealed that all four models exhibited strong structural validity. The fit indices for the classroom environment were as follows: *χ*^2^/*df* = 1.426, CFI = 0.991, GFI = 0.970, AGFI = 0.954, RMSEA = 0.032, and SRMR = 0.0255. For grit, the fit indices were: *χ*^2^/*df* = 1.126, CFI = 0.998, GFI = 0.984, AGFI = 0.972, RMSEA = 0.018, and SRMR = 0.0184. The fit indices for foreign language anxiety were: *χ*^2^/*df* = 1.703, CFI = 0.994, GFI = 0.988, AGFI = 0.971, RMSEA = 0.042, and SRMR = 0.0209. Finally, the fit indices for foreign language boredom were: *χ*^2^/*df* = 1.603, CFI = 0.992, GFI = 0.982, AGFI = 0.968, RMSEA = 0.038, and SRMR = 0.0213.

### 4.2 Descriptive and correlation analyses

Table 1 presents the descriptive statistics and Pearson correlation analysis data regarding the classroom environment, grit, foreign language anxiety, foreign language boredom, and foreign language academic achievement. According to the descriptive statistics, the mean scores for the classroom environment and grit were 3.312 (SD = 1.020) and 3.344 (SD = 1.095), respectively. The outcome reflects that the participants in the study perceived a relatively positive environmental experience in the classroom teaching scenario, where teacher support and student cohesion provided favorable perceptual feedback. Simultaneously, the participants demonstrated stable and sustained learning efforts in the process of foreign language acquisition, exhibiting high intrinsic interest and a willingness for continuous engagement in language learning, along with commendable grit qualities. Furthermore, the mean scores for foreign language anxiety and foreign language boredom were 2.984 (SD = 1.099) and 2.923 (SD = 1.095), respectively. Both variables fell within the moderately low range, indicating that participants experienced relatively less anxiety and boredom triggered by language learning tasks during their foreign language learning activities.

**Table 1:** Descriptive statistics and correlation analyses (*N* = 409).

Variables	Classroom environment	Grit	Foreign language anxiety	Foreign language boredom	Foreign language academic achievement	M(SD)
Classroom environment	–					3.312 (1.020)
Grit	0.566**	–				3.344 (1.095)
Foreign language anxiety	−0.490^**^	−0.526^**^	–			2.984 (1.099)
Foreign language boredom	−0.586^**^	−0.541^**^	0.495^**^	–		2.923 (1.095)
Foreign language academic achievement	0.592**	0.591**	−0.512**	−0.540**	–	

^**^p < 0.01.

The analysis presented in Table 1 indicates significant correlations among all variables. Notably, the classroom environment, grit, and foreign language academic achievement exhibited moderately strong positive correlations, with correlation coefficients of 0.592 and 0.591, respectively. The findings confirm that positive classroom environment and stable grit traits can effectively enhance learners’ foreign language academic achievement. Furthermore, the findings underscore the importance of fostering supportive classroom environment and cultivating grit to improve foreign language performance. Foreign language anxiety and boredom demonstrate moderate negative correlations with foreign language academic achievement, with correlation coefficients of −0.512 and −0.540, respectively. The relationship suggests that anxiety and boredom in foreign language learning can adversely affect learners’ academic performance. Meanwhile, there are moderate negative correlations between the classroom environment, grit, foreign language anxiety and boredom, with correlation coefficients of −0.490, −0.586, −0.526, and −0.541, respectively. It indicates that a more favorable classroom environment and a higher level of grit could reduce anxiety and boredom in foreign language learning. Consequently, it suggests that both the classroom environment and grit significantly influence the development of positive academic emotions and the alleviation of negative emotions in foreign language contexts.

### 4.3 Test for the hypothesized model

#### 4.3.1 The influence relationships between variables

**Figure 2:**
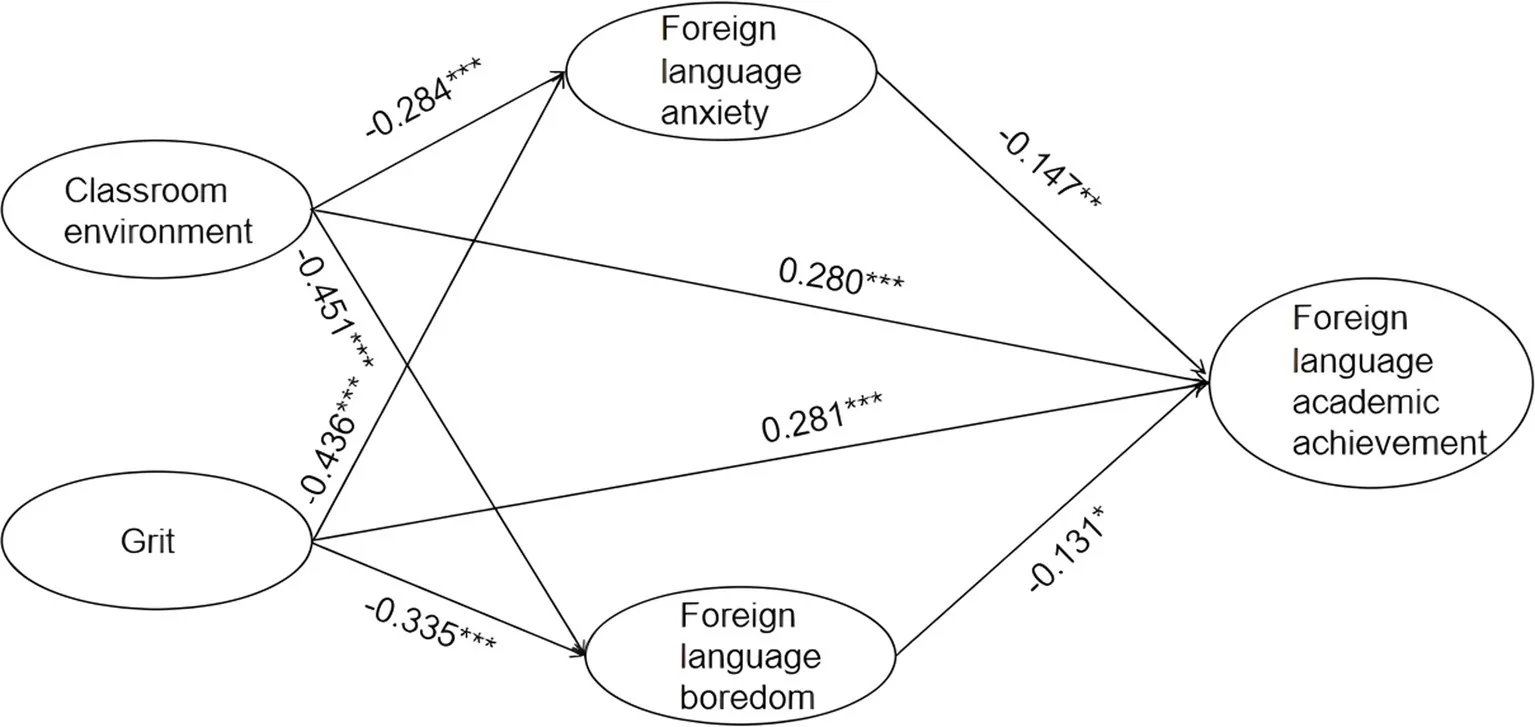
The influence relationships between variables. ^*^p < 0.05, ^**^p < 0.01, ^***^p < 0.001.

The hypothesized model was tested by Amos 24.0, yielding the following model fit indices: *χ*^2^/*df* = 1.184, CFI = 0.986, GFI = 0.916, AGFI = 0.905, RMSEA = 0.021, and SRMR = 0.0360. The results indicate a favorable model fit. The inferential statistical analysis of path coefficients between variables is presented in Table 2 and Figure 2. Both the classroom environment (*β* = 0.280, *p* < 0.001) and grit (*β* = 0.281, *p* < 0.001) exhibited significant positive predictive effects on foreign language academic achievement. Furthermore, the classroom environment demonstrated significant negative predictive effects on foreign language anxiety (*β* = −0.284, *p* < 0.001) and foreign language boredom (*β* = −0.451, *p* < 0.001). Grit also had a significant negative predictive effect on foreign language anxiety (*β* = −0.436, *p* < 0.001) and foreign language boredom (*β* = −0.335, *p* < 0.001). Notably, both foreign language anxiety (*β* = −0.147, *p* = 0.008) and foreign language boredom (*β* = −0.131, *p* = 0.026) demonstrated significant negative predictive effects on foreign language academic achievement. Based on the research findings, Hypothesis 1 and Hypothesis 2 of the study are supported.

**Table 2:** Summary of model regression coefficients.

Pathway	S.E.	C.R.	*p*	*β*
Classroom environment→foreign language academic achievement	0.067	4.321	***	0.280
Classroom environment→foreign language anxiety	0.072	−4.215	***	−0.284
Classroom environment→foreign language boredom	0.074	−6.795	***	−0.451
Grit→foreign language academic achievement	0.066	4.273	***	0.281
Grit→foreign language anxiety	0.073	−6.277	***	−0.436
Grit→foreign language boredom	0.070	−5.241	***	−0.335
Foreign language anxiety→foreign language academic achievement	0.053	−2.662	0.008	−0.147
Foreign language boredom→foreign language academic achievement	0.055	−2.224	0.026	−0.131

***p < 0.001.

#### 4.3.2 The mediating effect test

To explore the intrinsic mechanisms through classroom environment and grit on foreign language academic achievement, the study utilized the SPSS PROCESS macro (Model 4) to examine the parallel mediating effects of foreign language anxiety and foreign language boredom by taking repeated samples (5,000 bootstrap samples). First, the model test with the classroom environment as the independent variable demonstrated that foreign language anxiety and foreign language boredom played significantly partial mediating roles. The path analysis (as shown in Table 3) revealed that the classroom environment not only directly and positively predicted foreign language academic achievement (*β* = 0.343, *p* < 0.001), but also significantly and negatively predicted foreign language anxiety (*β* = −0.528, *p* < 0.001) and foreign language boredom (*β* = −0.630, *p* < 0.001). Both foreign language anxiety (*β* = −0.212, *p* < 0.001) and foreign language boredom (*β* = −0.202, *p* < 0.001) were identified as significant negative predictors of foreign language academic achievement. The Bootstrap mediation test results (as shown in Table 4) further corroborated the findings. The indirect effect of the classroom environment on foreign language academic achievement through foreign language anxiety was 0.112, with a 95% confidence interval (CI) of [0.065, 0.164], while the indirect effect through foreign language boredom was 0.127, with a 95% CI of [0.074, 0.186]. Notably, the confidence intervals for both paths do not encompass zero, indicating significant indirect effects. Furthermore, the mediating effect through foreign language boredom appears to be slightly stronger.

**Table 3:** Path analysis of the hypothesized model.

Pathway	*β*	S.E.	*t*	*p*	95% CI	
					Lower	Upper
Classroom environment→foreign language academic achievement	0.343	0.047	7.315	0.000	0.251	0.436
Classroom environment→foreign language anxiety	−0.528	0.047	−11.333	0.000	−0.620	−0.437
Foreign language anxiety→foreign language academic achievement	−0.212	0.041	−5.218	0.000	−0.291	−0.132
Classroom environment→foreign language boredom	−0.630	0.043	−14.607	0.000	−0.715	−0.545
Foreign language boredom→foreign language academic achievement	−0.202	0.044	−4.616	0.000	−0.288	−0.116
Grit→foreign language academic achievement	0.319	0.043	7.394	0.000	0.234	0.403
Grit→foreign language anxiety	−0.528	0.042	−12.474	0.000	−0.611	−0.445
Foreign language anxiety→foreign language academic achievement	−0.187	0.042	−4.510	0.000	−0.269	−0.106
Grit→foreign language boredom	−0.541	0.042	−12.974	0.000	−0.623	−0.459
Foreign language boredom→foreign language academic achievement	−0.229	0.042	−5.445	0.000	−0.312	−0.147

**Table 4:** The mediation analysis (Bootstrap = 5,000).

Independent variable	Effect type	Effect size	BootSE	95% CI lower	95% CI upper
Classroom environment	Total indirect effect	0.239	0.036	0.172	0.313
	Indirect effect of foreign language anxiety	0.112	0.025	0.065	0.164
	Indirect effect of foreign language boredom	0.127	0.029	0.074	0.186
Grit	Total indirect effect	0.223	0.033	0.160	0.292
	Indirect effect of foreign language anxiety	0.099	0.025	0.051	0.151
	Indirect effect of foreign language boredom	0.124	0.025	0.078	0.174

Secondly, the model test with grit as the independent variable demonstrated that foreign language anxiety and foreign language boredom played significantly partial mediating roles. Path analysis (as shown in Table 3) indicates that grit not only directly and positively predicts foreign language academic achievement (*β* = 0.319, *p* < 0.001), but also significantly and negatively predicts foreign language anxiety (*β* = −0.528, *p* < 0.001) and foreign language boredom (*β* = −0.541, *p* < 0.001). The results of the mediation analysis (as shown in Table 4) revealed that the indirect effect of grit on foreign language academic achievement through foreign language anxiety was 0.099, with a 95% CI of [0.051, 0.151], while the indirect effect through foreign language boredom was 0.124, with a 95% CI of [0.078, 0.174]. The confidence intervals for both pathways did not include zero, indicating significant indirect effects. Furthermore, the indirect path effect through foreign language boredom is relatively strong. Based on the research findings, Hypothesis 3 of the study is supported.

## 5 Discussion

### 5.1 Correlations between classroom environment, grit, foreign language anxiety, foreign language boredom, and foreign language academic achievement

The study found that classroom environment had a significant negative correlation with foreign language anxiety and foreign language boredom. The result indicates a closed relationship between the classroom environment and learners’ emotional states in the academic process. Specifically, a positive and uplifting classroom environment can effectively stimulate students’ positive emotions by improving teacher-student interaction patterns, enhancing peer collaboration, and optimizing classroom activity design. Conversely, a negative and oppressive classroom environment is likely to induce various negative emotions of students (Majumder and Beri, 2025). Furthermore, grit also exhibited a significant negative correlation with foreign language anxiety and foreign language boredom. It suggests that students with a high level of grit are more proactive in their foreign language learning process, actively addressing difficulties encountered in their studies, maintaining sustained engagement, and experiencing greater clarity in their classroom learning experiences. And their negative emotions would correspondingly decrease as well. Meanwhile, foreign language academic achievement is significantly and positively correlated with classroom environment and grit, while exhibiting a significant negative correlation with foreign language anxiety and boredom. The finding can be interpreted through the lens of the control-value theory: the positive classroom environment fosters conducive conditions for students’ learning activities, whereas grit equips students with the resilience to confront challenges and persist. These factors enhance students’ academic achievement. While foreign language anxiety and boredom diminish students’ sense of control and value identification regarding learning tasks, and undermine their interest and persistence, which ultimately negatively impacts their academic performance.

However, the findings of the study are clearly at odds with those of Khajavy et al. (2021). Khajavy et al. (2021) did not identify a correlation between grit and language achievement. The discrepancy may stem from their use of a domain-general grit scale. As Schmidt et al. (2019) noted, domain-general constructs often struggle to accurately measure and effectively predict behavior in specific contexts. While our study employs the L2-Grit Scale developed by Teimouri et al. (2022), which is more targeted and better reflects learners’ grit levels in language courses and aligns more closely with the actual needs of language research. The above reasons may explain the divergence between the conclusions of our study and those of Khajavy et al. (2021). Additionally, our findings diverge from those of Nasution et al. (2022). They reported a significant positive correlation between anxiety and learners’ English achievement. The discrepancy may be attributed to variations in measurement groups, learning stages, learner ages, educational levels, and language goals. These factors can influence the pathways and effects of anxiety on foreign language academic achievement. The subjects in Nasution et al. (2022) were students of grade 11. The anxiety may stimulate learning motivation, encourage them to actively identify and address their weaknesses, and enhance their foreign language learning performance. However, the subjects of our study comprise college students, whose age, stage of English learning, and goals differ markedly from those of high school students. Consequently, the mechanisms through which anxiety affects their foreign language academic achievement are also different. Furthermore, the research conducted by Dewaele et al. (2025) indicated that the relationship between foreign language anxiety and attitude/motivation fluctuated based on learners’ age which further supported the explanations provided in this paper for the observed discrepancies in the research. Moreover, the findings of the study differ from those of Masruroh et al. (2025). In their study involving 105 Indonesian junior high school students, they found only a weak negative correlation between foreign language anxiety and English achievement, which was not statistically significant. In contrast, our study revealed a moderate and significantly negative relationship between anxiety and foreign language academic achievement with Chinese learners as participants. Such inconsistent results can be explained from the perspective of native language–second language distance. From the standpoint of linguistic lineage and structural differences, Indonesian students’ native language is Indonesian, which belongs to a different language family than English but has incorporated numerous English loanwords at the lexical level. In contrast, Chinese and English share almost no commonalities in phonology, writing systems, or other aspects, representing a typical case of distant languages. Therefore, compared to Chinese learners, Indonesian learners may experience relatively less impact from linguistic distance during English learning, weakening the inhibitory effect of second language anxiety on English achievement, resulting in only a weak and non-significant correlation.

### 5.2 Direct pathways between classroom environment, grit, foreign language anxiety, foreign language boredom, and foreign language academic achievement

Consistent with the initial hypotheses of our study, the findings indicate that both the classroom environment and grit positively predict foreign language academic achievement. The results align with relevant research findings (Majumder et al., 2025; Botes et al., 2025). However, some studies have reported different results. For instance, Zheng and Feng (2023) argued that the classroom environment does not directly influence academic achievement but may exert an indirect effect through foreign language learning boredom. It might be because learners with greater autonomy and stronger foundational knowledge can benefit directly from a high quality classroom environment, which provides well-organized teaching content and efficient classroom interaction support. It facilitates learners’ knowledge internalization, enhances their learning efficiency and directly impacts their academic performance. However, for learners with relatively low autonomy and weaker foundational skills, classroom environment tends to indirectly influence academic achievement by internal psychological factors. It should be noted that since the Classroom Environment Scale developed by Peng and Woodrow (2010) measures students’ perceptions of the classroom environment through self-report, the discussion of classroom environment in this study primarily reflects participants’ subjective perceptions. Thus, the results are influenced by subjective factors such as individual cognition, learning experiences, and personal characteristics, and do not constitute a completely objective measurement of classroom environment features.

Furthermore, our study contrasts with the findings of Khajavy et al. (2021), which did not identify grit as a predictor of learners’ foreign language academic achievement. Firstly, it might be because the language domain-specific L2-Grit Scale we used provides a more accurate measure of learners’ effort and interest in foreign language learning contexts, elucidating grit’s predictive role more clearly in foreign language academic achievement. Secondly, the differences in research subjects between the two studies may also contribute to the discrepancy. Khajavy et al. (2021) examined Iranian students, while our study involved Chinese students. Variations in educational systems, foreign language learning environment, and academic evaluation methods may influence the functioning of grit and affect its predictive effect on foreign language academic achievement.

In addition, the study found that foreign language anxiety and foreign language boredom significantly predicted foreign language achievement, aligning with the findings of Li and Xing (2024). According to the control-value theory, academic emotions are important antecedent variables influencing subsequent learning behaviors and academic outcomes. As a negative academic emotion, foreign language anxiety alters learners’ cognitive allocation, engagement, and task performance quality (Liu et al., 2025), hindering the absorption, processing, and internalization of comprehensible input, suppressing language acquisition efficiency, and ultimately affecting learners’ foreign language academic performance. Furthermore, foreign language anxiety consumes limited working memory resources (Zheng and Cheng, 2018), thereby interfering with the entire process of language input reception, processing, and output, leading to reduced processing efficiency and lower accuracy and fluency in output, which continuously negatively impacts foreign language academic achievement. However, regarding the influence of foreign language boredom on foreign language academic achievement, the findings of this study differ from those of Dewaele et al. (2023b), who concluded that among three emotions (foreign language enjoyment, foreign language anxiety, and foreign language boredom), only foreign language anxiety effectively predicted foreign language academic achievement. The discrepancy may stem from the differences in the measurement indicators of academic achievement and the subjects between the two studies. Our study utilized latest final examination scores to assess academic achievement, whereas Dewaele et al. (2023b) employed participants’ latest foreign language test and final examination scores as measurement indicators. In short term testing situations, the influence of immediate anxiety may be more pronounced, potentially overshadowing the role of foreign language boredom. Furthermore, nearly one-third of the sample in Dewaele et al. (2023b) comprised secondary school students, while all participants in our study were university students. Learners at different educational stages have different emotional experiences. And the impact mechanisms through these experiences on academic achievement may be different which leads to the discrepancy in research findings.

### 5.3 The mediating effect of foreign language anxiety and foreign language boredom

It has been found that foreign language anxiety and foreign language boredom play partial mediating roles in the influence of the classroom environment on foreign language academic achievement. Specifically, positive classroom support environment can directly promote learners’ academic performance (Majumder et al., 2025) and provide learners with a clear instructional structure, a supportive atmosphere, and a cooperative learning context. These factors effectively enhance learners’ sense of control and perceived value in foreign language learning tasks, and simultaneously reduce their perceptions of foreign language anxiety and boredom. As two prevalent negative academic emotions, foreign language anxiety can consume cognitive processing resources and interfere with the language learning process (Liu et al., 2025), whereas foreign language boredom can diminish learning engagement and intrinsic motivation (Lyu, 2025). Both of the two emotions negatively impact foreign language academic achievement.

Similarly, foreign language anxiety and foreign language boredom partially mediate the relationship between grit and foreign language academic achievement. Learners with higher L2 grit inherently possess greater learning resilience and more clearly defined goal orientations. Their L2 grit can directly enhance their foreign language proficiency (Fathi et al., 2026). Additionally, learners with higher levels of grit tend to maintain positive perceptions of their foreign language learning abilities, which enables them to exert better control over tasks and appreciate the value of foreign language learning. The resilience effectively mitigates foreign language anxiety (Li and Dewaele, 2021) and foreign language boredom (Lyu, 2025), which promotes learners’ academic achievement. The findings confirm the independent facilitating effects of classroom environment and grit on learners’ academic success, and also support the core proposition of the control-value theory, which posits that distal environmental triggers can induce learners’ emotions through proximal control-value appraisals and affect academic performance. As distal triggers, classroom environment and personality traits can elicit learners’ emotions through proximal control and value appraisals, and further influence academic achievement.

Meanwhile, the study found that in the above mediation analysis, the mediating effect of foreign language boredom was slightly higher than that of foreign language anxiety. It might partly be attributed to the distinct emphases of foreign language boredom and anxiety in terms of their triggering mechanisms, pathways of influence, and intensity of impact on learning behaviors. Foreign language boredom often arises from learners’ insufficient perception of task value or a lack of sense of control, leading to cognitive fatigue. The emotional state may exert prolonged negative effects on learning motivation and resilience (Macklem, 2015). In contrast, although foreign language anxiety also interferes with the effective allocation of cognitive resources (Liu et al., 2025), its impact might be more focused on the immediate stage of language processing and tends to diminish after learners receive positive feedback or engage in emotional regulation, exhibiting situational and temporary characteristics. Therefore, over the long term of foreign language learning, foreign language boredom might more persistently trigger avoidance behaviors, reducing learners’ actual opportunities for language practice. Thus, it exerts a slightly stronger mediating role in the pathway through which classroom environment and grit influence foreign language academic achievement, ultimately resulting in a marginally greater mediation effect compared to foreign language anxiety.

## 6 Conclusion

The study integrated the classroom environment (external context), grit (internal personal trait), foreign language academic emotions (mediating variable), and foreign language academic achievement within a unified analytical framework. It systematically examined the mediating role of foreign language academic emotions in the relationships between the classroom environment/grit, and foreign language academic achievement. The results indicate that foreign language academic emotions play significant mediating roles, serving as a crucial factor that connects the external learning context with individual characteristics and influences academic achievement. The findings illuminate the intrinsic pathways of influence between the variables, underscore the critical role of emotions in the foreign language learning process, and expand research perspectives within the framework of the control-value theory. Additionally, the research findings provide empirical evidence for optimizing foreign language teaching practices and classroom environment, fostering learners’ positive personality traits and academic emotions, and enhancing foreign language academic achievement.

Admittedly, there are limitations in the study. First, the research focused exclusively on students from three universities in China, which might raise concerns about the representativeness and generalizability of the findings. Thus, future research should use a stratified random sample to control for the demographic differences between participants. Second, regarding the measurement of foreign language academic achievement, the study utilized the latest final exam scores as measurement indicator. It may not adequately capture the dynamic and comprehensive nature of learners’ foreign language outcomes and overall language proficiency. Future studies should utilize the integrating multiple data sources, such as teacher evaluations, language application performance, and periodic assessments to develop a more comprehensive and multi-dimensional evaluation system for foreign language academic achievement. Third, the study integrated the three dimensions into an overall construct for analysis, failing to further explore the differential effects of the sub-dimensions of classroom environment on learners’ foreign language academic emotions and achievement. Future research could disentangle these three sub-dimensions, conduct precise dimension-specific analyses, compare the varying influences of different classroom environment dimensions, thereby enriching the findings on classroom environment and foreign language learning.

## Data Availability

The raw data supporting the conclusions of this article will be made available by the authors, without undue reservation.

## References

[ref2] Botes E. Azari Noughabi M. Amirian S. M. R. Greiff S. (2025). New wine in new bottles? L2 grit in comparison to domain-general grit, conscientiousness, and cognitive ability as a predictor of language learning. J. Multiling. Multicult. Dev. 46, 2451–2466. doi: 10.1080/01434632.2023.2294120

[ref3] Cheng Y. Ping H. (2025). An empirical investigation into teacher-student interactional silence in college translation classroom. Educ. Rev. USA 9, 546–551. doi: 10.26855/er.2025.05.010

[ref5] Dai K. Wang Y. (2024). Enjoyable, anxious, or bored? Investigating Chinese EFL learners’ classroom emotions and their engagement in technology-based EMI classrooms. System 123:103339. doi: 10.1016/j.system.2024.103339

[ref7] Dewaele J. M. Botes E. (2025). How the effects of foreign language enjoyment, anxiety, boredom and peace of mind on attitude/motivation change with skill level, academic achievement and age: a moderated mediation model. Int. J. Appl. Linguist. 35, 2010–2025. doi: 10.1111/ijal.12749

[ref8] Dewaele J. M. Botes E. Greiff S. (2023b). Sources and effects of foreign language enjoyment, anxiety, and boredom: a structural equation modelling approach. Stud. Second. Lang. Acquis. 45, 461–479. doi: 10.1017/s0272263122000328

[ref9] Dewaele J. M. Botes E. Meftah R. (2023a). A three-body problem: the effects of foreign language anxiety, enjoyment, and boredom on academic achievement. Annu. Rev. Appl. Linguist. 43, 7–22. doi: 10.1017/s0267190523000016

[ref10] Dewaele J. M. Guedat-Bittighoffer D. Dat M. A. (2025). Foreign language enjoyment overcomes anxiety and boredom to boost oral proficiency in the first year of English foreign language learning. Int. J. Appl. Linguist. 35, 152–167. doi: 10.1111/ijal.12607

[ref11] Dikmen M. (2021). EFL learners’ foreign language learning anxiety and language performance: a meta-analysis study. Int. J. Contemp. Educ. Res. 8, 206–222. doi: 10.33200/ijcer.908048

[ref12] Dorman J. P. Fisher D. L. Waldrip B. G. (2006). “Classroom environment, students’ perceptions of assessment, academic efficacy and attitude to science: a LISREL analysis,” in Contemporary Approaches to Research on Learning Environments: Worldviews, eds. Fisher D. Khine M. S. (Singapore: World Scientific).

[ref13] Duckworth A. L. Peterson C. Matthews M. D. Kelly D. R. (2007). Grit: perseverance and passion for long term goals. J. Pers. Soc. Psychol. 92, 1087–1101. doi: 10.1037/0022-3514.92.6.1087, 17547490

[ref14] Fathi J. Pawlak M. Saeedian S. Ghaderi A. (2026). Exploring factors affecting foreign language achievement: the role of growth mindset, self-efficacy, and L2 grit. Lang. Teach. Res. 30, 3528–3559. doi: 10.1177/13621688241227603

[ref15] Fraser B. J. Treagust D. F. Dennis N. C. (1986). Development of an instrument for assessing classroom psychosocial environment at universities and colleges. Stud. High. Educ. 11, 43–54. doi: 10.1080/03075078612331378451

[ref16] Fraser B. J. (2002). “Learning environments research: yesterday, today and tomorrow,” in Studies in Educational Learning Environments: An International Perspective, eds. S. C. Goh and M. S. Khine. (Singapore: World Scientific).

[ref17] Harpaz G. Vaizman T. Yaffe Y. (2024). University students’ academic grit and academic achievements predicted by subjective well-being, coping resources, and self-cultivation characteristics. High. Educ. Q. 78, 192–211. doi: 10.1111/hequ.12455

[ref18] Horwitz E. K. Horwitz M. B. Cope J. (1986). Foreign language classroom anxiety. Mod. Lang. J. 70, 125–132. doi: 10.1111/j.1540-4781.1986.tb05256.x

[ref19] Hu L. Bentler P. (1995). “Evaluating model fit,” in Structural Equation Modeling: Concepts, Issues, and Applications, ed. Hoyle R. H. (London: Sage).

[ref20] Hu L. Bentler P. (1999). Cutoff criteria for fit indexes in covariance structure analysis: conventional criteria versus new alternatives. Struct. Equ. Model. 6, 1–55. doi: 10.1080/10705519909540118

[ref21] Hu X. Zhang X. McGeown S. (2024). Foreign language anxiety and achievement: a study of primary school students learning English in China. Lang. Teach. Res. 28, 1594–1615. doi: 10.1177/13621688211032332

[ref22] Huang F. Zhang H. (2025). The relationship between boredom and second language achievement: a multilevel meta-analysis. Stud. Second. Lang. Acquis. 47, 488–511. doi: 10.1017/s0272263125000142

[ref24] Jiang Y. Dewaele J. M. (2019). How unique is the foreign language classroom enjoyment and anxiety of Chinese EFL learners? System 82, 13–25. doi: 10.1016/j.system.2019.02.017

[ref25] Jin F. Gu C. Li Y. (2024). Trait emotional intelligence and foreign language performance: associations with academic self-efficacy and foreign language anxiety. Front. Educ. 9:1419328. doi: 10.3389/feduc.2024.1419328

[ref26] Khajavy G. H. MacIntyre P. D. Hariri J. (2021). A closer look at grit and language mindset as predictors of foreign language achievement. Stud. Second. Lang. Acquis. 43, 379–402. doi: 10.1017/s0272263120000480

[ref27] Lee J. S. (2022). The role of grit and classroom enjoyment in EFL learners’ willingness to communicate. J. Multiling. Multicult. Dev. 43, 452–468. doi: 10.1080/01434632.2020.1746319

[ref28] Li C. Dewaele J. M. (2021). How classroom environment and general grit predict foreign language classroom anxiety of Chinese EFL students. J. Psychol. Lang. Learn. 3, 86–98. doi: 10.52598/jpll/3/2/6

[ref29] Li C. Dewaele J. M. Hu Y. (2023). Foreign language learning boredom: conceptualization and measurement. Appl. Linguist. Rev. 14, 223–249. doi: 10.1515/applirev-2020-0124

[ref30] Li C. Feng E. Li S. (2025). Boredom and achievement in L2 learning: a meta-analysis. Appl. Linguist. Rev. 16, 2373–2399. doi: 10.1515/applirev-2024-0266

[ref31] Li C. Huang J. Li B. (2021). The predictive effects of classroom environment and trait emotional intelligence on foreign language enjoyment and anxiety. System 96:102393. doi: 10.1016/j.system.2020.102393

[ref32] Li C. Li W. (2023). Anxiety, enjoyment, and boredom in language learning amongst junior secondary students in rural China: how do they contribute to L2 achievement? Stud. Second. Lang. Acquis. 45, 93–108. doi: 10.1017/s0272263122000031

[ref33] Li Z. Xing L. (2024). Foreign language anxiety, enjoyment, and boredom among Chinese secondary students: a control-value theory approach. Humanit. Soc. Sci. Commun. 11:556. doi: 10.1057/s41599-024-03049-7

[ref34] Li C. Yang Y. (2024). Domain-general grit and domain-specific grit: conceptual structures, measurement, and associations with the achievement of German as a foreign language. Int. Rev. Appl. Linguist. Lang. Teach. 62, 1513–1537. doi: 10.1515/iral-2022-0196

[ref35] Liu M. (2023). English classroom anxiety, learning style and English achievement in Chinese university EFL students. Sustainability 15:13697. doi: 10.3390/su151813697

[ref36] Liu H. Li T. Zheng H. Li Y. Fan J. (2025). Exploring the relationship between students’ language learning curiosity and academic achievement: the mediating role of foreign language anxiety. Behav. Sci. 15:1133. doi: 10.3390/bs15081133, 40867490 PMC12383800

[ref37] Liu G. L. Zhang Y. Zhang R. (2024). Examining the relationships among motivation, informal digital learning of English, and foreign language enjoyment: an explanatory mixed-method study. ReCALL 36, 72–88. doi: 10.1017/s0958344023000204

[ref38] Luan W. Quan J. (2025). Interplay among classroom environment, grit, and enjoyment in shaping feedback-seeking behavior in L2 writing. Behav. Sci. 15:584. doi: 10.3390/bs15050584, 40426362 PMC12109084

[ref39] Luo Z. Xiong Y. (2025). Is anxiety always harmful? An exploration of the impact of language anxiety on EFL learning motivation, EFL competence and EFL academic performance. BMC Psychol. 13:674. doi: 10.1186/s40359-025-03017-z, 40598665 PMC12220092

[ref40] Lyu J. (2025). A mixed-methods approach to the psychological predictors of boredom in second language learning: mindfulness, grit, and self-regulation. Front. Psychol. 12:1609330. doi: 10.3389/fpsyg.2025.1609330, 40919595 PMC12412256

[ref41] Ma L. Liu L. Luo S. Liu J. (2025). Exploring urban-rural differences in foreign language enjoyment, anxiety, boredom, and burnout among Chinese secondary students. J. Multiling. Multicult. Dev. 46, 2498–2516. doi: 10.1080/01434632.2023.2298691

[ref42] Ma W. Yang W. Bu Q. (2024). Interconnected factors in EFL engagement: classroom climate, growth mindset, and achievement goals. Front. Psychol. 15:1353360. doi: 10.3389/fpsyg.2024.1353360, 39529721 PMC11550954

[ref43] MacIntyre P. D. (1999). “Language anxiety: a review of the research for language teachers,” in Affect in Foreign Language and Second Language Learning: A Practical Guide to Creating a Low-Anxiety Classroom Atmosphere, ed. Young D. J. (Boston, MA: McGraw-Hill).

[ref44] Macklem G. L. (2015). Boredom in the Classroom: Addressing Student Motivation, Self-Regulation, and Engagement in Learning. Cham: Springer.

[ref45] Majumder P. Beri N. (2025). Systematic review of classroom climate and its influence on English language achievement. Multidiscip. Rev. 8:2025134. doi: 10.31893/multirev.2025134, 41669154

[ref9001] Majumder P. Beri N. Biswas S. (2025). Classroom climate and absenteeism: modeling English achievement mediated by engagement. *International Journal of Evaluation & Research in Education*, 14, 4432–4442. doi: 10.11591/ijere.v14i6.35795

[ref46] Masruroh D. Aridah A. Rusmawaty D. (2025). Foreign language anxiety and its impact on English achievement: a study of gender variations in EFL learners. Voices Engl. Lang. Educ. Soc. 9, 86–95.

[ref47] Mikami H. (2024). Revalidation of the L2-grit scale: a conceptual replication of Teimouri, Y., Plonsky, L.,& Tabandeh, F. (2022). L2 grit: passion and perseverance for second-language learning. Lang. Teach. 57, 274–289. doi: 10.1017/s0261444822000544

[ref48] Nasution R. F. Manuas M. J. Ali M. I. Pabur H. E. Tatipang D. P. (2022). How anxiety affect English achievement?: a correlational study of Indonesian EFL learners. J. Engl. Cult. Lang. Lit. Educ. 10, 321–337. doi: 10.53682/eclue.v10i2.5540

[ref49] Pawlak M. Derakhshan A. Mehdizadeh M. Kruk M. (2025). Boredom in online English language classes: mediating variables and coping strategies. Lang. Teach. Res. 29, 509–534. doi: 10.1177/13621688211064944

[ref50] Pawlak M. Kruk M. Zawodniak J. Pasikowski S. (2020). Investigating factors responsible for boredom in English classes: the case of advanced learners. System 91:102259. doi: 10.1016/j.system.2020.102259

[ref51] Pawlak M. Kruk M. Zawodniak J. Pasikowski S. (2022). Examining the underlying structure of after-class boredom experienced by English majors. System 106:102769. doi: 10.1016/j.system.2022.102769

[ref52] Pekrun R. (2006). The control-value theory of achievement emotions: assumptions, corollaries, and implications for educational research and practice. Educ. Psychol. Rev. 18, 315–341. doi: 10.1007/s10648-006-9029-9

[ref53] Pekrun R. Elliot A. J. Maier M. A. (2006). Achievement goals and discrete achievement emotions: a theoretical model and prospective test. J. Educ. Psychol. 98, 583–597. doi: 10.1037/0022-0663.98.3.583

[ref54] Pekrun R. Goetz T. Titz W. Perry R. P. (2002). Academic emotions in students’ self-regulated learning and achievement: a program of qualitative and quantitative research. Educ. Psychol. 37, 91–105. doi: 10.1207/s15326985ep3702_4

[ref55] Pekrun R. Marsh H. W. Elliot A. J. Stockinger K. Perry R. P. Vogl E. . (2023). A three-dimensional taxonomy of achievement emotions. J. Pers. Soc. Psychol. 124, 145–178. doi: 10.1037/pspp0000448, 36521161

[ref56] Peng J. Woodrow L. J. (2010). Willingness to communicate in English: a model in the Chinese EFL classroom context. Lang. Learn. 60, 834–876. doi: 10.1111/j.1467-9922.2010.00576.x

[ref57] Schermelleh-Engel K. Moosbrugger H. Müller H. (2003). Evaluating the fit of structural equation models: tests of significance and descriptive goodness-of-fit measures. Methods Psychol. Res. Online 8, 23–74. doi: 10.23668/psycharchives.12784

[ref58] Schmidt F. T. Fleckenstein J. Retelsdorf J. Eskreis-Winkler L. Möller J. (2019). Measuring grit: a German validation and a domain-specific approach to grit. Eur. J. Psychol. Assess. 35, 436–447. doi: 10.1027/1015-5759/A000407

[ref59] Schreiber J. B. Nora A. Stage F. K. Barlow E. A. King J. (2006). Reporting structural equation modeling and confirmatory factor analysis results: a review. J. Educ. Res. 99, 323–338. doi: 10.3200/joer.99.6.323-338

[ref60] Schumacker R. E. Lomax R. G. (1996). A Beginner’s Guide to Structural Equation Modeling. Mahwah, NJ: Lawrence Erlbaum Associates.

[ref61] Shimray R. Wangdi T. (2025). Boredom in online foreign language classrooms: antecedents and solutions from students’ perspective. J. Multiling. Multicult. Dev. 46, 288–303. doi: 10.1080/01434632.2023.2178442

[ref62] Teimouri Y. Goetze J. Plonsky L. (2019). Second language anxiety and achievement: a meta-analysis. Stud. Second. Lang. Acquis. 41, 363–387. doi: 10.1017/S0272263118000311

[ref63] Teimouri Y. Plonsky L. Tabandeh F. (2022). L2 grit: passion and perseverance for second language learning. Lang. Teach. Res. 26, 893–918. doi: 10.1177/1362168820921895

[ref65] Wang X. Hui L. (2024). Buoyancy and engagement in online English learning: the mediating roles and complex interactions of anxiety, enjoyment, and boredom. System 125:103418. doi: 10.1016/j.system.2024.103418

[ref66] Wu Y. Kang X. (2023). The relationship between academic boredom and EFL achievement: examining the mediating role of behavioral engagement. J. Lang. Teach. 3, 1–10. doi: 10.54475/jlt.2023.002

[ref67] Xiao J. Zhang Y. (2024). Exploring the effect of language-related academic emotions on foreign language achievement: a systematic review and meta analysis. Int. J. Engl. Lang. Stud. 6, 43–56. doi: 10.32996/ijels.2024.6.2.7

[ref68] Yamashita T. (2018). Grit and Second Language Acquisition: Can Passion and Perseverance Predict Performance in Japanese Language Learning? Massachusetts: University of Massachusetts Amherst.

[ref69] Zhao X. Sun P. P. Gong M. (2024). The merit of grit and emotions in L2 Chinese online language achievement: a case of Arabian students. Int. J. Multiling. 21, 1653–1679. doi: 10.1080/14790718.2023.2202403

[ref70] Zhao X. Wang D. (2023). Grit, emotions, and their effects on ethnic minority students’ English language learning achievements: a structural equation modelling analysis. System 113:102979. doi: 10.1016/j.system.2023.102979

[ref71] Zheng Y. Cheng L. (2018). How does anxiety influence language performance? From the perspectives of foreign language classroom anxiety and cognitive test anxiety. Lang. Test. Asia. 8:13. doi: 10.1186/s40468-018-0065-4

[ref9002] Zheng R. and Feng E. (2023). The correlations between perceived classroom environment, foreign language learning boredom and EFL achievement: a full mediation model. *Chinese Journal of Applied Linguistics*, 46, 605–624. doi: 10.1515/CJAL-2023-0407

